# Determining Multi-Layer Factors That Drive the Carbon Capability of Urban Residents in Response to Climate Change: An Exploratory Qualitative Study in China

**DOI:** 10.3390/ijerph15081607

**Published:** 2018-07-29

**Authors:** Jia Wei, Hong Chen, Ruyin Long

**Affiliations:** 1 School of Economics and Finance, Xi’an Jiaotong University, Xi’an 710061, China; 2 School of Management, China University of Mining and Technology, Xuzhou 221116, China; longruyin@cumt.edu.cn

**Keywords:** carbon capability, driving factor, grounded theory, urban resident

## Abstract

The active promotion of carbon abatement to mitigate global climate change and protect the environment and public health has become the international consensus. The carbon capability is a key index for measuring the potential reduction of the carbon emissions by urban residents, and thus encouraging residents to exhibit normal and autonomous low-carbon behavior has become an important issue. In this study, based on grounded theory, data from in-depth interviews were encoded at three levels to identify the multi-layer factors that drive the carbon capability of urban residents, and we constructed a theoretical model for policy intervention. The results showed that individual factors, organizational factors, social factors, and social demographic variables were the main variables that affected the carbon capability, and utility experience perception was the main intermediary variable that affected the carbon capability. There was an obvious gap between utility experience perception and carbon capability. Low carbon selection cost was an internal situational variable that regulated the relationship between these factors, and the policy situation and technical situation were external situational variables. There were two-way effects on the carbon capability and utility experience perception. Thus, we explored these driving factors and the role of the carbon capability model. The results of this study may facilitate targeted policy thinking and the development of an implementation path for government in order to formulate effective guiding policies to enhance the carbon capability of urban residents.

## 1. Introduction

Global climate change is one of the most serious challenges facing mankind in the 21st century, where it influences the natural environment and the health and survival of humans and other organisms [1,2]. According to the “Global Climate Risk Index 2018”, more than 524,000 people have died worldwide as a direct result of over 11,000 extreme weather events [3]. It is well known that carbon dioxide emissions are the main cause of global warming. In addition, other pollutants such as sulfur dioxide and nitrogen dioxide are extremely harmful to public health [4,5,6,7]. Therefore, in order to protect the environment and public health, carbon emissions need to be reduced as soon as possible. The carbon emission intensity is most closely related to energy consumption. The U.S. Energy Information Agency estimated that global energy-related carbon emissions increased by 21% between 2005 and 2017, mainly from Asian countries such as China and India [8]. Energy-related carbon emissions are expected to remain relatively stable in most regions by 2019, but emissions from China are still increasing [8]. In June 2015, the Chinese government issued a statement regarding the intended nationally determined contributions (INDC), with a promise to reduce carbon emissions per unit of GDP by 60% by 2030 compared with 2005 [9]. In a context characterized by the comprehensive implementation of energy saving and emission reduction measures, energy consumption by industry has begun to achieve negative growth, but the consumption of domestic energy is increasing annually in the industrial sector, and the proportion of energy consumed has increased rapidly since 2010 [10]. According to the data in the Energy Statistics Yearbook, the per capita energy consumption by urban residents is much higher than that by rural residents, where the difference is as high as 3.5 times [10]. In addition, according to the “National New Urbanization Plan 2014–2020” issued by the State Council, the urbanization rate for China’s resident population will increase from 52.6% in 2013 to 60% in 2020 [11], which means that the energy consumption by urban residents will continue to account for most of the overall energy consumed by residents. In addition, at present, China is still in a period of rapid industrialization and urbanization. Due to the sustained growth of the economy, life in the context of urbanization and the continuous changes in transportation modes mean that the energy consumption by urban residents and the resulting carbon emissions will increase further. Understanding how to exploit the potential low-carbon emission reductions by residents is an important issue for the development of low-carbon economy. Residents are the third largest producers of emissions after the government and enterprises, and thus the motivation and ability to reduce carbon emissions is important for social progress.

In this context, previous studies have explored the “carbon capability” concept to measure the motivation and potential of residents regarding reductions in their carbon emissions [12,13,14]. The carbon capability refers to the “capability of individuals to make wise judgments about low carbon and take effective measures.” Previous studies have emphasized the need to consider knowledge, decisions, individual behaviors, and collective behaviors. Evidently, clear judgments and the effective implementation of low-carbon behaviors are core elements of the carbon capability, but the capacity for low-carbon behavior is not equal to the carbon capability itself. According to the classical theory of capability research, i.e., iceberg theory, capability is not confined only to knowledge and skills that comprise the superficial part above the sea level, i.e., the value concept. Instead, the motivation that lurks in the deep part below sea level is the key for distinguishing individual differences in capability [15,16,17]. The capabilities of urban residents that can be observed easily, such as knowledge, skill, and behavior, are explicit features, but the elements lurking below sea level, such as low-carbon value concepts, are motivations that are deeply rooted in residents. These motivations are very important for understanding, evaluating, and improving the low-carbon capability. Wei et al. proposed the concept of the carbon capability [14]. The carbon capability of urban residents comprises an advanced set of abilities related to the overall process, which ranges from establishing the low carbon value concept, mastering the skills of low carbon identification, making wise low carbon choices, taking effective low carbon actions, and having a low carbon influence. The carbon capability is widely acknowledged as having five dimensions: carbon value, carbon identification, carbon choice, carbon mobility, and carbon impact. The concept of carbon capability maturity was subsequently proposed and it was suggested that an important premise for building guiding policies for developing the carbon capability is exploring the key factors responsible for driving the carbon capability, but these factors have been considered little in previous studies [15]. Several studies have focused on the carbon capability concept and standard measurements, but few have conducted specific analyses of the factors that affect the carbon capability. Thus, it is necessary to identify the drivers of carbon generation and development in order to guide and enhance the ability of urban residents to reduce carbon emissions effectively. Essentially, we need to identify the underlying psychological processes related to the carbon capability and understand how these psychological processes contribute to the transformation of low carbon cognition into low carbon behavior. In addition, we need to formulate effective intervention policies to shape and enhance the carbon capability, and ensure the acceptability and effectiveness of these interventions. These issues have not been addressed in previous theoretical studies.

Therefore, in this study, we aimed to describe, illustrate, and interpret the factors that drive the carbon capability of urban residents. We explored the main drivers of changes in the carbon capability of urban residents and the mechanisms related to these factors. We identified possible external intervention policies to promote the carbon capability of urban residents and the intervention path for these policies, as well as providing a theoretical basis and policy reference to help relevant government agencies formulate effective intervention policies.

## 2. Literature Review

Obtaining deep insights into the factors that influence the carbon capability of urban residents is important for exploring effective intervention policies. However, there have been few specific analyses of the factors that affect the carbon capability and only a few related studies have considered the capacity for low-carbon behavior. The low carbon consumption ability is regarded as the antecedent variable for low carbon consumption behavior. For example, Mi et al. regarded the “low carbon behavior ability” as a factor that influences “low carbon behavior willingness,” which directly affects the low-carbon energy consumption behavior of urban residents [18]. Given that the carbon capability is an actual ability, it is necessary to consider its characteristics and the factors that influence it. The factors that influence this ability are genetic, environmental, and non-intellectual (emotional processes, will processes, temperament, and personality) [19]. In addition, given that knowledge and skills are the cognitive basis of competence, and individual behavior is the carrier of capacity, then low-carbon knowledge/skills are the cognitive basis of low-carbon capability, and low-carbon consumer behavior is the result of carbon capability behavior. Therefore, it is necessary to review the theory of low-carbon knowledge/skills, low-carbon consumption behavior, and related research.

Many theoretical and empirical studies have investigated low-carbon consumption behavior, green consumption behavior, and other types of energy consumption behavior from different perspectives. These variables are not completely consistent with the low carbon consumption behavior in terms of their connotation and extension, but the conclusions of these studies provide a useful reference for low carbon consumption behavior. Previous studies have explored the key variables related to the mechanism of low-carbon consumption behavior in urban residents. However, studies of external factors such as subjective norms and social responsibility consciousness have mostly considered endogenous psychological factors, including the environmental attitude, environmental values, and other variables [20], whereas few have investigated the key psychological variables related to deeper factor-personality traits. Personality is the core of individual beliefs, values, and attitudes. Many studies have shown that personality traits have significant impacts on environmental behavior [21,22]. Thus, differences in personality affect individual environmental attitudes and environmental behaviors [23]. Low carbon consumption behavior is an environmental behavioral concept, and thus personality traits may also affect the low carbon consumption behavior of individuals [24]. In addition, demographic variables can be employed to analyze the differences in the low-carbon consumption behaviors of urban residents [25,26,27,28], where these variables include gender, age, marital status, education, occupation, and income. In general, theoretical studies have focused on two areas: the factors that influence low-carbon consumption behavior and the internal mechanism involved; and intervention policies and their effectiveness at promoting low-carbon consumption behavior [29,30,31]. The related factors include individual characteristics (such as age, occupation, income, and educational background), endogenous psychological factors, and exogenous situational factors. However, most previous studies focused on either the factors that influence low-carbon consumption behavior or intervention policies, whereas few have attempted to integrate both perspectives by exploring the determinants and intervention policies at the same time [32].

In summary, studies of the variables that affect the carbon capability of urban residents are still very rare. There is a lack of in-depth research into the mechanisms and policy guidance path for the factors that influence the carbon capability. Therefore, based on previous research, we investigated the main factors that drive the carbon capability of urban residents and explored the policy guidance path.

## 3. Research Methods and Data Sources

### 3.1. Research Methods

**Figure 1:**
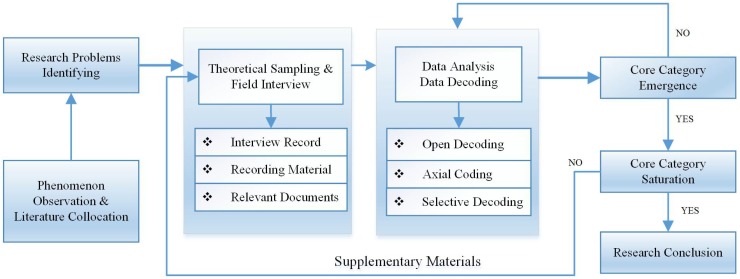
Encoding process and overall qualitative research method.

The carbon capability of urban residents is not completely clear at present, and thus it is necessary to conduct exploratory qualitative research. Therefore, the exploratory method employed for data analysis in this study was mainly based on grounded theory. Grounded theory was proposed in 1967 [33] and it is now regarded as “the most influential research paradigm in modern social science and at the forefront of the qualitative research revolution” [34,35,36]. The original analysis of data in grounded theory was mostly based on written data. In this study, we recorded in-depth interviews with representative urban residents. The core idea of grounded theoretical analysis is continuous comparative analysis, which is embodied in the contents of a document before and after analysis, and between the data. The concepts and category elements related to the research goal can be found via continuous comparative analysis. Attributes can be generalized and the corresponding theory may be constructed from the bottom to the top. The specific steps in grounded theoretical analysis mainly include open coding, axial coding, and selective coding, as shown in Figure 1.

Based on the process described above, we determined the main factors related to the carbon capability of urban residents, especially the factors that drive it and the mechanism responsible, before establishing the relationships between various concepts and category elements, and finally constructing a theoretical framework as a model of the mechanisms that drive the carbon capability of urban residents. The overall research process is illustrated schematically in Figure 1.

### 3.2. Data Collection

The first process in qualitative research is data collection. In this study, representative urban residents from the sample area were interviewed in depth to obtain first-hand information to understand their cognition and attitude regarding low carbon consumption. In order to explore the main factors that drive the carbon capability of urban residents and establish a policy guidance path, it is necessary to identify the barriers perceived by urban residents related to the implementation of low-carbon behavior.

In previous qualitative studies, face-to-face interviews have often been used to obtain the required information. In this study, in addition to the usual face-to-face interviews, we used the QQ chat platform WeChat chat platform to conduct interviews. QQ chat, which is short for Tencent QQ, is an internet-based instant messaging software. At present, QQ is available for Microsoft Windows, OS X, Android, iOS, Windows Phone, and other mainstream platforms. QQ supports online chat, video calls, point-to-point breakpoint file continuation, shared files, network hard disk, custom panel, QQ mailbox, and other functions, and it can be connected to a variety of communication terminals. WeChat is also a free messaging service for smart terminals. WeChat supports voice messages, video, images, and text messages, which can be sent free of charge (where a small amount of the network traffic is consumed) over the network across communication operators and across operating system platforms. In this study, voice call, video call, and messaging platforms were used for interviews. Participants were free to choose their preferred platform for the interviews.

This type of online interview has the following advantages: the interviewees do not have to meet with the interviewers directly, which is more convenient in terms of time and space; the interviewees do not feel constrained and they may answer more freely and truthfully; the conversation is not easily influenced by the verbal and behavioral language of the interviewer; and the answers can be more considered and logical. Therefore, we conducted in depth, one-to-one interviews via the QQ and WeChat chat platforms online. In the interviews, we used a method based on questions in a focused interview, where the interviewer set up a participatory dialogue and guided the interviewees to focus on a specific subject from their own point of view. Each in-depth interview lasted about 1 h and sufficient time was allowed for reflection by the interviewees. Before the formal interview, the interviewer first explained the subject and the issues considered with the interviewee via the message platform. The interview was based on the principles of openness, interaction, and confidentiality. According to the requirements of grounded theory, the interviews involved no presuppositions and paradigms, but simple interview outlines were specified in advance (see Table 1) to improve the efficiency of the interviews.

In the interview, further tracked questions focused on the problems in Table 1 and the concept categories were captured in order to explore the interviewee’s inner psychology as deeply as possible to extend the interview content. For example, when asked what measures the government should implement to promote the carbon capability, if the interviewee mentioned monitoring, then the interviewer asked: “Do you think government supervision can promote carbon enhancement? Can you specify how the government should monitor it?”

### 3.3. Sample Selection

The interview is the main source of qualitative research data, and thus the conclusions obtained, so the interviewees should be selected carefully. Therefore, the interviewees needed to have some understanding of the carbon capability, and low-carbon related concepts and behaviors. Representative urban residents were identified by combining theoretical sampling with stratified sampling, where the representative residents had some understanding of low-carbon consumption and a certain level of educational knowledge [37]. We limited the interviewees by age to 20–45 years and they comprised urban residents with undergraduate educational levels or above, as well as having some understanding of low-carbon consumption and behavior [37]. We also considered gender, age, occupation, and other variables to ensure that a reasonable sample structure was obtained in accordance with the real situation. After theoretical sampling and stratified sampling, two specific interviews were conducted. The sample size was determined based on the principle of theoretical saturation, i.e., sampling until the new samples no longer provided new important information. After 38 subjects had been interviewed, the answers given in the last three interviews assessed by content analysis were unable to add new information to the study, so the content was saturated. According to this process, 35 participants were identified. The data showed that the sample matched the diversity requirements for the qualitative study, including different genders, ages, regions, and occupational backgrounds. The basic information for the interviewees is shown in Table 2.

## 4. Category Extraction and Model Construction

After sorting the interview records, two-thirds of the interview records were randomly selected for root coding analysis and other one-third were tested for grounded theoretical saturation. In order to ensure the reliability and validity of the study, the grounded coding program developed by Strauss and Corbin was strictly applied in the grounded coding process [38]. In addition, in order to avoid the effects of personal prejudice on the coding results and to improve the objectivity and scientific validity of the coding, we combined personal coding and expert consultation in this study.

### 4.1. Open Coding

In the encoding process, open coding or first-level coding is also known as open login or level 1 login. During the open coding process, we tried to use the original words of the interviewees as much as possible in order to name the concepts directly or to extract the related concepts, thereby eliminating the effects of prejudice by the individual coder as much as possible. We invited five related professional researchers to organize the data from the interviews and collected 1021 expressions related to the “carbon capability of urban residents.” In order to identify the factors that strongly influence the carbon capability of urban residents, we excluded relatively simple and vague answers from the interview records, and we finally obtained more than 800 original sentences and the corresponding initial concepts. The number of initial concepts was high and there was a certain degree of crossover between them, so the initial concepts with a repetition frequency of more than five times were selected for categorization, and individual contradictory initial concepts were also eliminated at the same time. Open coding commenced after the first interview. In the first interview, some concepts were sorted and the correlations and differences between these concepts were analyzed to yield some final categories. Next, the second interview was conducted by targeting the problems found in the coding process and the sorting of the concept categories. It should be noted that not everyone was interviewed twice. After the first interview, we open coded the collected data. If questions or doubts remained regarding some of the participants, we contacted them to arrange a second interview. This process was required because the numbers of concepts and categories determined by coding were relatively high, and the related concepts and categories were repeated in the coding process, so the interview could not proceed and coding could not reach the next level. Table 3 presents the conceptualization and categorization processes based on the original interview records. The categorization results comprised factors related to the carbon capability of urban residents. Considering limitations on space for this article, we only list representative primitive record statements and the initial concepts for each category.

### 4.2. Axial Coding

In the encoding process, axial coding or secondary encoding is also known as associative login or axis login. Axial coding is a key component of grounded theory analysis. The original codes for the objects or concepts generated in the open encoding process are the major focus of the analysis in axial coding. Axial coding follows a coding paradigm comprising: “reason-phenomenon scene-mediation conditions-action/interaction strategy-results” [39]. This paradigm combines every concept identified in the open coding process to form a main category, as well as developing and testing the relationship among the concepts. It should be noted that the use of an analysis “paradigm” is important because it provides a perspective for viewing the original concept and a heuristic analysis tool to help organize the systematic decentralization of the original concept.

Axial coding involves the discovery and establishment of various relationships between conceptual categories. In axial coding, researchers only analyze one category at a time by exploring the correlations with this category and analyzing potential correlations between each category at the conceptual level. Therefore, this process is called the “axis” or “spindle.” After analyzing the correlations between each group of categories, we need to recognize the level of the category in the group, i.e., the main category and sub-category, before establishing the relationships between the principal category and sub-categories by continual comparative analysis. The main category identification process (axial coding process) is shown in Table 4.

### 4.3. Selective Coding

In the coding process, selective coding or three-level coding is also known as core login or selective login. Selective coding is based on applying spindle coding to further identify and systematically analyze the relationships between categories and categories. Selective coding is a form of systematic analysis for identifying the core category from the main category and systematically establishing the connections between the core category and other categories. In the continual comparative analysis process, the core category must be repeatedly identified as dominant in most categories and it should clearly describe the relationships between most categories. Most categories can be included in a theoretical framework with good coverage. After in-depth mining, the typical relationships between the main categories determined by selective encoding are shown in Table 5.

### 4.4. Saturation Test

Theoretical saturation testing refers to the further development of a category feature based on the use of no additional data as the criterion for stopping sampling [40]. After sorting the interview records, two-thirds of the interview records were randomly selected for root coding analysis and one-third were tested for grounded theoretical saturation. The coding analysis of the remaining one-third of the interview records was consistent with the above description, where we conducted open coding, axial coding, and selective coding. Information saturation was determined by continuous comparative analysis. The saturation test was complete and the qualitative analysis ended when no new conceptual categories could be found. In this study, the results showed that the categories in the model were sufficiently developed, and no new categories or relationships were found for the main carbon capability categories of urban residents and no new constituent factors were formed within the main categories. Therefore, we consider that the analysis performed in this study was theoretically saturated.

### 4.5. Model Construction

**Figure 2:**
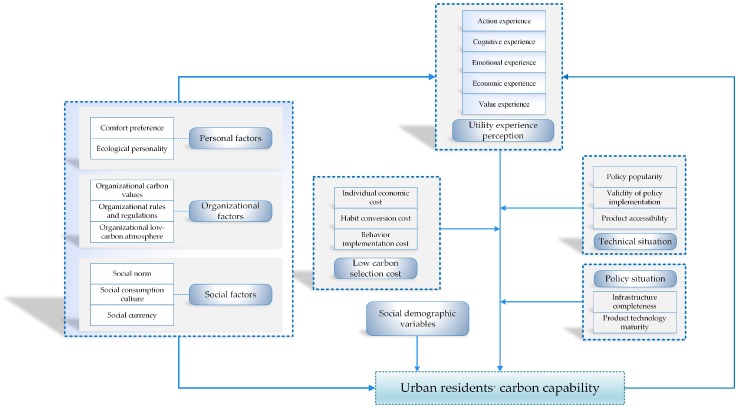
Comprehensive theoretical model of the mechanisms that drive the carbon capability of urban residents.

Based on the analysis described above, we identified the core drivers of the carbon capability in urban residents and analyzed their interactions. The specific driving factors comprised the individual factors, organizational factors, social demographic variables, utility experience perception, policy situation, low carbon choice cost, and technological situation. In addition, based on the typical relationship structure (Section 4.3), we determined the core category for the multi-layer factors that drive the carbon capability of urban residents and the policy guidance path. We then developed a new theoretical framework comprising the factors that drive the carbon capability in a theoretical model of the mechanisms that drive the carbon capability of urban residents, as shown in Figure 2.

## 5. Discussion

According to the variables (individual factors, organizational factors, and social factors) that affect the carbon capability, we identified two key drivers based on the in-depth interviews: ecological personality and social currency. These two factors have been considered little in previous studies. (1) Ecological personality. Based on the in-depth interviews with residents and experts, we found that the personality traits of residents can affect the construction and development of their carbon capability, and the acceptance of the ecological environment concept can actually promote the implementation of low-carbon consumption behavior. According to previous studies, personality traits will affect the implementation of individual low-carbon consumption behavior. The ecological and environmental consequences of changes in consumption patterns by ordinary consumers are ultimately limited. We cannot be overly optimistic until the current consumption pattern changes because the deterioration of the ecological environment will continue [1,41,42]. In particular, the ecological personality is not a traditional psychological personality, but instead it can be regarded as an ecological trait. Thus, from the perspective of an ecological civilization, the traditional personality connotation can no longer fully explain individual behavioral changes in an ecological situation, and the specific connotation of personality is changing constantly due to the change from an industrial civilization to an ecological civilization. Therefore, the ecological personality trend is evolving into a necessity [43]. The ecological personality combines a unique and stable thinking mode, and behavioral style based on the ecological concept and the fusion of the human social mentality and natural ecology [44], which have important effects on the construction and promotion of the carbon capability. (2) Social currency. This factor conforms with Bandura’s social learning theory. According to Bandura’s social learning theory, human behavior is the result of information processing activities and their interactions with internal factors and environmental factors [45]. Thus, low carbon consumption behavior is a consequence of individual personality, psychological consciousness, and external social factors. In our daily lives, we share various types of information with friends, such as via Weibo (this “MicroBlog” is a social network platform in China for share briefs and real-time messages via an attention mechanism), and the circle of friends allows others to express themselves. If people receive a message that attracts attention and conveys their likes and dislikes regarding low-carbon behavior, then the likelihood of choosing and implementing low-carbon consumption will be greatly increased [15,46]. A message that makes someone feel unique is a form of social currency. Social currency is derived from the concept of social media economics, which measures the tendency of users to share brand-related content. In a study of “content sharing” via Internet social networking, Magdol and Bessel concluded that when people communicate with others, they are not only trying to communicate information because “people also want to spread certain information about themselves” [47]. Thus, people subconsciously want to “label” themselves by talking to others about what they see as their ideal self, which can be funny, intelligent, strong, beautiful, or rich. Our in-depth interviews demonstrated that social currency is factor that can drive the carbon capability of urban residents.

There is a bidirectional interaction between utility experience perception and carbon capability of urban residents. Utility experience perception is a decisive factor that affects the development of the carbon capability. Thus, when the individual’s perception of the outcome of a certain capability tends to be negative, then they consider that the capability does not have any benefit for them, so there is no possibility of carbon capability construction and development. Very few studies have analyzed the impact of utility experience perception on the carbon capability. The meaning of utility experience perception and its relationship with the carbon capability of urban residents was defined mainly according to the results of the in-depth interviews. According to the contents of the interviews, most residents suggested that low-carbon behaviors can reduce economic expenses, bring spiritual satisfaction, protect the environment, and obtain other outcomes. Therefore, we defined the low-carbon consumption behavior as a carrier of the carbon capability, which can lead to utility experience perception, and affect the construction and development of the carbon capability of urban residents. This effect can be positively promoted or reversed, and thus there is a bidirectional interaction between utility experience perception and the carbon capability of urban residents.

Similar to the “attitude behavior” gap, there is a significant gap between utility experience perception and the carbon capability of urban residents. If an individual has a positive utility experience perception, the carbon capability does not necessarily increase. In the interviews, we obtained some data that supported the perception of the utility experience and the generation of the carbon capability in a similar manner to the relationship between behavioral will and behavior. Few studies have investigated the relationships between environmental attitudes (will/intention) and environmental behavior, but most showed that pro-environmental attitudes lead to environmentally friendly behavior [48]. Some studies found incomplete agreement between environmental attitudes (will/intention) and environmental behavior, but they demonstrated a synergy between environmental attitudes (will/intention) and environmental behavior. For example, a study of the energy use attitude and actual energy use behavior of residents in three regions of Finland found a gap between the two [49]. The energy use attitude of the residents tended to favor low-carbon or green behaviors, but other factors that affected the comfort of life meant that the actual rate of change in behavior was very slow [49]. Thus, similar to the gap between “attitude and behavior,” we found a clear gap between the construction of the carbon capability of urban residents and the perception of development and the utility experience, which requires further study.

The low carbon selection cost is an internal situational factor related to the carbon capability of urban residents and by acting as an internal regulatory variable, it exerts a regulatory effect between utility experience perception and the carbon capability. The in-depth interviews showed that many urban residents have not implemented low-carbon behavior, mainly because they feel that low-carbon products are more expensive and they find them inconvenient. If the implementation of a specific low-carbon consumption behavior is very difficult and it causes discomfort in one’s everyday life, as well as requiring more time and effort, then the possibility of its implementation will be very low, which may counteract the perceived positive utility experience that has been generated. Therefore, the low carbon selection cost acts as an internal regulatory variable with a regulatory effect between utility experience perception and the carbon capability. The individual will evaluate the costs and benefits of an individual behavioral choice before making a decision, and the individual will reject the choice when the cost exceeds a certain level. The cost for an individual choosing a low-carbon behavior includes the cost of selecting and implementing low-carbon consumption behavior, but also the costs of transforming traditional consumption habits and implementing a behavioral choice. These internal situational factors influence the implementation of low-carbon consumption patterns.

The technological situation and the policy situation are external situational factors that affect the carbon capability of urban residents, and as external regulatory variables, they have regulatory interactive effects between the utility experience perception and the carbon capability. According to behavioral theory, attitudes regarding individual environmental awareness, purchasing, and consumption behavior will be affected by the product price, performance, and convenience [50]. Therefore, when individuals make a low-carbon consumption decision, their behavior will be affected by the price, performance, and convenience of the product. More comprehensive considerations of low-carbon products should consider their technological maturity, availability, convenience, and other effects. In addition, the degree of policy intervention, validity, and infrastructure improvement also influence individual low-carbon lifestyle choices to a great extent [51]. According to the in-depth interviews, many urban residents do not use low-carbon travel modes because the infrastructure is not adequate and many other studies reached a similar conclusion [29,37]. Therefore, adequate infrastructure is an important requirement for low-carbon behavior. Moreover, the government should improve the relevant system policies and increase policy support. Based on the results of the in-depth interviews, we identified technological situational factors and policy situational factors as external situational factors that affect the carbon capability of urban residents, such as the technological maturity of products, accessibility, and infrastructure completeness. We also found a regulatory interaction between utility experience perception and the carbon capability.

## 6. Conclusions and Policy Implications

Based on the grounded theory, we showed that individual factors, organizational factors, social factors, utility experience perception, policy situation, low carbon selection cost, technical situation, and social demographic variables are the main factors that drive the carbon capability of urban residents. The individual factors mainly include the ecological personality and comfort preference. The organizational factors include organizational values, organizational system norms, and low carbon atmosphere. The social factors mainly include social consumption culture, social norms, and social currency. The policy situational factors mainly include the degree of policy popularization and the validity of policy implementation. The cost of low carbon selection mainly includes personal economic cost, habit transformation cost, and behavior implementation cost. The technological situational factors mainly include product technological maturity, product availability, and infrastructure completeness. The social demographic variables include individual statistical characteristics, family statistical characteristics, organizational statistical characteristics, and urban statistical characteristics.

The theoretical contributions of this study are as follows. (1) We systematically explored the driving factors that affect the urban carbon capability and identified the leading factors related to the carbon capability (individual factors, organizational factors, and social factors), the perception of utility experience, internal situational factors (low carbon selection cost), and external situational factors (policy situation and technical context). (2) Based on this multi-layer exploration of the factors that influence the individual, organization, and society, we constructed a theoretical model of the factors that drive the carbon capability of urban residents, including the mechanism responsible for forming the carbon capability of urban residents, and we suggested that the perception of utility experience is an important individual factor. Organizational factors and social factors are the three main variables that influence the carbon capability, which is regulated by situational factors. The perception of utility can affect the generation and development of the carbon capability, but it can also have the reverse effect on the carbon capability. (3) The multi-layer driving factors identified in this study contribute greatly to carbon capability research. The ecological personality among the individual factors and social currency among the social factors have rarely been investigated in previous studies.

**Figure 3:**
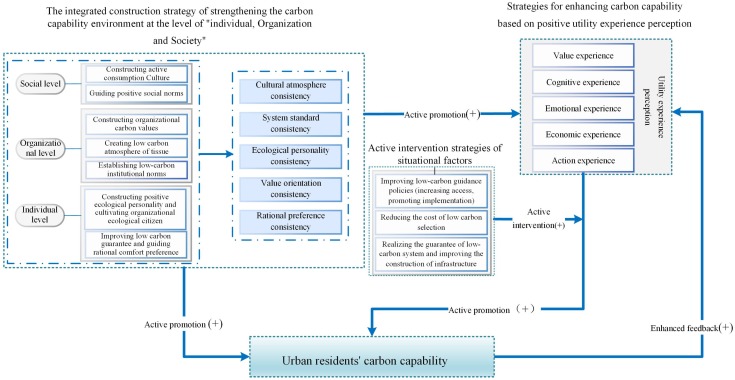
“Advanced cycle” for the construction of the carbon capability of urban residents, with positive utility experience perception as the core.

The conclusions of this study are also important for management practice. In order to effectively guide the development of the carbon capability of urban residents and implement low-carbon behavior, the three aspects shown in Figure 3 should be considered when formulating guidance policies.

(1) The integrated construction of a strategy for increasing the carbon capability at the levels of the individual, organization, and society. At the social level, the government should build a positive consumer culture and guide positive social norms. At the organizational level, it is necessary to build the organizational carbon value, create a low-carbon attitude in organizations, and establish low-carbon system norms. At the individual level, the government should build a positive ecological personality and cultivate ecological citizens, as well as improving low-carbon security and guiding rational and comfortable preferences. In order to achieve consistent effects on the individual, organization, and society in the terms of the cultural atmosphere, institutional norms, ecological personality, value orientation, and rational preferences, we need to address the following issues. (1) Governmental aspects such as improving appropriate laws, regulations, and standards of environmental protection, establishing the concept of unified economic development and environmental protection, promoting balanced development, providing ecological education to all people, cultivating ecological citizens, improving the system for environmental information disclosure, enhancing the supervision system, and implementing “carbon-free” operations. (2) Actively encourage low-carbon behavior by enterprises, such as promoting a low-carbon management model, establishing low-carbon guidance system norms, maximizing environmental benefits, improving the technological level of low-carbon products and services, and reducing direct/indirect carbon emissions as much as possible while still satisfying the requirements in terms of comfort for residents. (3) Positively promoting a low-carbon lifestyle among the public, such as encouraging a green diet, green clothing, green living, green travel, and building a low-carbon community.

(2) Strategies for enhancing the carbon capability based on positive utility experience perception. The carbon capability has a positive influence on utility experience perception, and utility experience perception has a positive effect on the carbon capability, so positively and efficiently enhancing the carbon capability will further enhance and consolidate the carbon capability of urban residents. The government can motivate urban residents by rewarding innovative low-carbon applications, subsidizing low carbon products, and rewarding advanced low-carbon individuals/enterprises in order to stimulate urban residents and help urban residents to build their own carbon capability. The government should spread information to the public about how climate warming is caused by high carbon emissions as well as its impacts on physical and mental health, thereby enhancing the perception of the public. The government should improve the purchase channels and provide guidelines for low-carbon products to ensure that urban residents have positive economic experiences when selecting low-carbon products, thereby enhancing their economic perception of these products. For example, on the consumer side, subsidies should be provided for goods such as energy-saving lamps and new energy automobiles, and the low-carbon preferences of urban residents must be cultivated. Furthermore, by growing the economy, environmental protection, value, and active experiences related to low carbon, we can increase the positive cognition of the carbon capability among residents and help them to build and enhance their own carbon capability.

(3) Situational factors have significant effects on promoting the utility-related perception of the carbon capability. Positive intervention strategies related to situational factors are required, such as improving economic policies to promote energy conservation and reduce emissions, as well as considering the popularization and implementation of policies. First, the government should innovate methods for popularizing low carbon knowledge and technology by explaining the specific regulations and implementation rules for policies to residents in various forms, and by implementing low-carbon guidance policies that are closely related to the interests of residents. Second, these strategies may include improving the energy saving and emissions reduction investment mechanism and reducing the costs of low carbon selection, improving the maturity of low-carbon technology, implementing low-carbon product projects and improving the low carbon product purchase channel, and constructing adequate infrastructure and increasing its effective supply. In particular, it is necessary to establish unified green product classification standards, certification bodies, and maturity marking systems. In addition, in terms of the infrastructure supply, the government should try to improve the modernization of the infrastructure, promote regional infrastructure development, and accelerate the development of infrastructure with a green low carbon cycle, as well as conducting regular maintenance and upgrading to ensure that the basic living needs of residents are met.

## Figures and Tables

**Table 1:** Outline of the open-ended interview.

Carbon Capability Comprised an Advanced Set of Capabilities, Which Range from Establishing the Low Carbon Value Concept to Mastering the Skills of Low Carbon Identification, Making Wise Low Carbon Choices, Taking Effective Low Carbon Actions, and Having a Low Carbon Influence.	
Interview theme	Main content outline
Basic information	Gender, age, income level, education, occupation, family structure, city, etc.
Carbon capability cognition	♦ What do you think of low carbon consumption? Is low carbon consumption necessary or not? ♦ In what ways do you think the carbon capability can be measured and can it be specified in the context of everyday life? ♦ Do you or your family, friends, colleagues have carbon capability? Do they have anything in common?
Driving factors	♦ In your opinion, what are the main factors that affect the carbon capability? ♦ What do you think you need to do to transform low-carbon awareness into practical behavior? ♦ What channels do you think you or your family use to acquire low-carbon related knowledge and skills? ♦ Do you pay attention to publicity and education regarding low carbon consumption and low carbon lifestyle? Does this propaganda have a positive effect on you? What type of publicity or education do you think is more effective at enhancing the carbon capability of most people? ♦ Do you think that increasing low carbon consumption awareness would make people more likely to implement it in practice? ♦ What do you think are the main obstacles and motivations that affect your choices in terms of low-carbon products or services? Are you affected by these factors every time you make a choice? If so, can you rank the importance of these factors? If not, can they be specified in terms of your actual life? ♦ Will your words and deeds affect low carbon consumption by the people around you? If so, what factors do you think inspired this ability? If not, what are the constraints? In addition, will you be affected by the low-carbon behavior of the people around you?
Intervention policies to enhance carbon capability	♦ In your view, how can we develop the carbon capability and change people to “low-carbon” lifestyle and consumption patterns from “high-carbon” behaviors? ♦ What do you think the government should do to maintain progress?

**Table 2:** Statistical information for the interviewees.

Attribute		Number of People	Percent (%)
Location	Beijing	10	28.57
	Guangdong	10	28.57
	Jiangsu	15	42.86
Interview mode	Face-to-face interview	20	57.14
	Online interview	15	42.86
Gender	Male	18	51.43
	Female	17	48.57
Occupation	Students (graduate students and above)	6	17.14
	Educational and scientific research, professional, and technical personnel	8	22.86
	Enterprises and institutions, managers	8	22.86
	Business, Services and others	13	37.14
Age	20–30 years	13	37.14
	31–45 years	12	34.29
	Above 45 years	10	28.57

**Table 3:** Process and results of open coding.

Raw Data Statement (Representative Statement)	Category
Comfort and convenience are of the utmost importance, and I find it hard to accept the sacrifice of comfort.	Comfort preference
I think some green enthusiasts are more likely to have carbon awareness and this must have something to do with their personality. Just like some people can be particularly sensitive to environmental issues, such as when they encounter haze, they feel it immediately and others become dull.	Ecological personality
The carbon capability must be cultivated. Mastering the carbon capability in our daily lives can also save costs, protect the environment, and benefit future generations (cognitive, economic, and value experience). I think carbon awareness is very fashionable, eye-catching, and cool, such as Tesla cars and cycling (emotional and action experience).	Utility experience perception
My family thinks about saving money to some extent. The low-carbon value depends mainly on the company and its leadership, but the overall value of the company is very important and it may affect me.	Organizational carbon values
It depends on our company’s regulation and whether it had a low-carbon orientation. If there is a requirement not to waste electricity, such as printing the paper on both sides, then I will also follow low-carbon behaviors at work, but if this is not the case, it depends on personal habits.	Organizational rules and regulations
If my boss and colleagues are low carbon-oriented and this is the overall atmosphere, then I may be affected.	Organizational low-carbon atmosphere
Many civil servants and celebrities say that they want a low-carbon society, but I am not convinced and I think they are doing it for show. No one will do it (low carbon). What’s the point of doing it? Let’s go with the flow.	Social norm
It has something to do with consumption habits and consumer culture. Chinese tradition prefers extravagance and large wedding banquets. Now, the consumer climate has changed. No one is proud of saving. Instead, you feel that you are stingy and have no (low carbon) atmosphere at all.	Social consumption culture
I care a lot about relationships and if low carbon gives me more credibility in front of my friends, then I am willing to do it. It is what you say and sometimes it is just a kind of conversation.	Social currency
Many people do not choose low-carbon products because they are likely to be poorly understood, and individual economic capabilities cannot be supported. If you can reduce the cost of living and be willing to improve the carbon capability, then it finally depends on the economic conditions.	Individual economic cost
The biggest obstacle is that people have formed habits where they are simply unwilling and unlikely to change. Even if some people do change, after a period of time they may return to their original state because they are accustomed to certain behaviors.	Habit conversion cost
I think that comfort and convenience are most important, so I would only prefer low-carbon behavior if it is convenient. I think (low-carbon) barriers may affect convenience and they are driven by the deterioration of the living environment.	Behavior implementation cost
There are no garbage collection devices on the roadside and rubbish is often placed in a trash bin because we do not know how to classify it. Some facilities need to be changed, such as smart homes and new energy car charging piles.	Infrastructure completeness
The quantity, quality, and popularity of the optional low-carbon products are not sufficient. There are only few brands that can be trusted or at least I have not heard of others.	Product technology maturity
I do not know low-carbon products or where to buy them. If I cannot buy it at any supermarket, it would be hard for me to accept it. After all, consumer goods are for convenience.	Product accessibility
I have rarely come into contact with low-carbon propaganda, such as the occasional billboard by the roadside. The state policy is not clear. I have heard of low-carbon consumption, but I do not know what it means.	Policy popularity
The implementation of the policy is not sufficiently strong. I feel that low carbon is empty talk, which no one will implement. It is mostly propaganda, such as big advertisements, and there is no consideration of the effects of publicity or its pertinence.	Valid implementation of policy
Most older people and other people who have status and rights will not choose low-carbon behaviors.	Age
I think it is easier for women to implement low-carbon behaviors.	Gender
Literate people may be more concerned. Most people have a general level of education and they do not know what low-carbon behavior means.	Record of formal schooling
People who have status and rights may not choose low-carbon behaviors. It depends mainly on occupation and income.	Occupation
It still depends on the economic conditions. If I have enough money, I may buy a Tesla car. If you have money, you must enjoy your life; otherwise, you will choose low-carbon behaviors.	Income level
It depends on the enterprises where you work. For example, state-owned enterprises and foreign enterprises may be more responsive to state propaganda, but some private enterprises may not have the same attitude.	Unit property
People aged over 40 or 50 years who have social status, or officials, may not choose low-carbon behavior because it is not necessary.	Job level
People who own a house might not choose low-carbon behaviors, but most who rent homes are not concerned.	Housing type
Children and old people pay more attention to shopping. Health is the most important thing and low-carbon behavior is not the main consideration.	Family structure
Large cities such as Beijing, Shanghai, and Guangzhou may be exposed to more ideas. Policy pilots are also being implemented in these places, so it is expected that the prevalence of low-carbon consumption will be slightly higher. I expect that small cities and rural areas will not know about low-carbon behaviors.	Urban characteristics

**Table 4:** Process and results of axial coding.

Fundamental Category	Corresponding Subcategory	Connotation of Category Relationship
Personal factors	Comfort preference	The comfort preference of urban residents is an individual factor that affects the carbon capability.
	Ecological personality	The ecological personality of urban residents is an individual psychological factor that affects the carbon capability.
Utility experience perception	Utility experience perception	The utility experience (cognitive, emotional, value, economic, and operational) perceived by individuals as a result of actual low-carbon behavior can affect the likelihood of low-carbon behavior and the construction of the carbon capability.
Organizational factors	Organizational carbon values	The values of the organizations to which urban residents belong will affect the individual’s low-carbon values and behaviors, and thus the factors with influence at the organizational level.
	Organizational rules and regulations	The institutional norms of the organizations to which urban residents belong will affect the individuals’ low carbon values and behaviors, and thus the factors with influence at the organizational level.
	Organizational low-carbon atmosphere	The low carbon atmosphere of the organization to which the urban residents belong will influence the individual’s low carbon values and low carbon behavior, and thus the factors with influence at the organizational level.
Social factors	Social norm	Social norms will affect the construction of the carbon capability of urban residents, and thus they are social level factors.
	Social consumption culture	The social consumption culture, such as comparing and flaunting, will affect the construction of the carbon capability of urban residents, and thus they are social level factors.
	Social currency	Social conversation, status, etc. are social factors that affect the carbon capability.
Low carbon selection cost	Individual economic cost	Personal economic level, standard of living, and considerations of self-interest will affect the cost of choosing low-carbon consumption by urban residents.
	Habit conversion cost	Consumption habits, living habits, and other habits will affect the cost of low-carbon consumption by urban residents.
	Behavior implementation cost	Behavioral convenience and maintenance costs will affect the cost of choosing low-carbon consumption for urban residents.
Technical situation	Infrastructure completeness	Some factors such as imperfect infrastructure and a poor recycling network will affect the selection of low-carbon consumption by urban residents, and thus these factors belong to the category that affects the construction of the carbon capability.
	Product technology maturity	Factors such as a low number of product varieties and immature technology will influence whether urban residents select low-carbon consumption, and thus these factors belong to the category that affects the construction of the carbon capability.
	Product accessibility	Factors such as easy access and selectivity will affect the choice of low-carbon consumption by urban residents, and thus these factors belong to the category that affects the construction of the carbon capability.
Policy situation	Policy popularity	The popularity of policies will affect the institutional situation and the construction of the carbon capability of urban residents.
	Validity of policy implementation	The valid implementation of policy will affect the institutional situation and the construction of the carbon capability of urban residents.
Social demographic variables	Individual statistical variables	Age, gender, education, occupation, income level, etc.
	Organizational work variables	Unit nature, job level, etc.
	Family statistical variables	Residential type, family structure, etc.
	Urban statistical variables	Urban characteristics

**Table 5:** Selective coding results.

Typical Relational Structure	Connotation of Relationship Structure
Utility experience perception → carbon capability	Utility experience perception is an internal driving factor related to capacity building, and utility experience perception directly determines whether an individual will build and develop this ability.
Carbon capability → Utility experience perception	The carbon capability can enhance the utility experience perception and have positive effects.
Individual factors → Utility experience perception → Carbon capability	The individual’s preference for comfort and ecological personality traits will determine their perception of the outcome of a certain capability. The ability to have a sense of satisfaction with all aspects, such as cognition, emotion, action, and economy will determine whether to build the capability.
Organizational factors → Utility experience perception → Carbon capability	The carbon values, institutional norms, and low-carbon atmosphere in an individual’s organization will affect the perception of the outcome of a given ability, i.e., whether the ability can lead to satisfaction with all aspects, such as cognition, emotion, action, and economy, and then to decide whether to build that capacity.
Social factors → Utility experience perception → Carbon capability	Social norms, social consumption culture, social money, and other social factors will affect an individual’s perception of the outcome of a given ability, i.e., whether an ability can lead to satisfaction with all aspects of one’s own cognition, emotion, action, and economy, and then to decide whether to build that capacity.
Low carbon selection cost ↓ Utility experience perception → Carbon capability	Low carbon selection cost is an internal situational condition related to carbon capability building and development. It affects the relationship between utility experience perception and the carbon capability path.
Technical situation ↓ Utility experience perception → Carbon capability	Technological situational factors comprise the external constraints on the construction and development of the carbon capability. As a regulating variable, the technological situational factors influence the perception of utility experience in the relationship between the carbon capability path and the relationship between the technological situational factors and the carbon capability path.
Policy situation ↓ Utility experience perception → Carbon capability	Policy situational factors comprise the external constraints on the construction and development of the carbon capability. As regulatory variables, policy situational factors influence the intensity and direction of the relationship between the perception of utility experience and the ability of carbon capability.
Social demographic variables → Carbon capability	Social demographic variables have significant direct impacts on the carbon capability, and social demographic variables directly determine the individual carbon capability.
